# Evasion of Early Antiviral Responses by Herpes Simplex Viruses

**DOI:** 10.1155/2015/593757

**Published:** 2015-03-30

**Authors:** Paula A. Suazo, Francisco J. Ibañez, Angello R. Retamal-Díaz, Marysol V. Paz-Fiblas, Susan M. Bueno, Alexis M. Kalergis, Pablo A. González

**Affiliations:** ^1^Millennium Institute on Immunology and Immunotherapy, Departamento de Genética Molecular y Microbiología, Facultad de Ciencias Biológicas, Pontificia Universidad Católica de Chile, 8331010 Santiago, Chile; ^2^INSERM UMR1064, 44093 Nantes, France; ^3^Departamento de Inmunología Clínica y Reumatología, Escuela de Medicina, Facultad de Medicina, Pontificia Universidad Católica de Chile, 8331010 Santiago, Chile

## Abstract

Besides overcoming physical constraints, such as extreme temperatures, reduced humidity, elevated pressure, and natural predators, human pathogens further need to overcome an arsenal of antimicrobial components evolved by the host to limit infection, replication and optimally, reinfection. Herpes simplex virus-1 (HSV-1) and herpes simplex virus-2 (HSV-2) infect humans at a high frequency and persist within the host for life by establishing latency in neurons. To gain access to these cells, herpes simplex viruses (HSVs) must replicate and block immediate host antiviral responses elicited by epithelial cells and innate immune components early after infection. During these processes, infected and noninfected neighboring cells, as well as tissue-resident and patrolling immune cells, will sense viral components and cell-associated danger signals and secrete soluble mediators. While type-I interferons aim at limiting virus spread, cytokines and chemokines will modulate resident and incoming immune cells. In this paper, we discuss recent findings relative to the early steps taking place during HSV infection and replication. Further, we discuss how HSVs evade detection by host cells and the molecular mechanisms evolved by these viruses to circumvent early antiviral mechanisms, ultimately leading to neuron infection and the establishment of latency.

## 1. Introduction

Herpesviruses are frequently found in humans, although their prevalence significantly varies depending on ethnicity, sex, and geographical location of individuals, among others [1–5]. Currently, eight Herpesviridae family members are known to infect humans: herpes simplex viruses (HSV) -1 and -2 (HSV-1, HHV-1 and HSV-2, HHV-2, resp.), varicella zoster virus (VZV, HHV-3), Epstein Barr (EBV, HHV-4), cytomegalovirus (CMV, HHV-5), human herpesvirus 6 (HHV-6), human herpesvirus 7 (HHV-7), and Kaposi sarcoma-associated virus (KSV or HHV-8). All herpesviruses harbor large genomes encoding >70 genes and share the capacity to establish lifelong persistent infections in the host ([6] and NCBI).

Human infection with herpes simplex viruses (HSVs) traces far back, even before the intercontinental migration of our ancestors, as proposed by recent phylogenetic analyses [7]. Symptomatic manifestations of HSVs have been described as early as 400 BC and these viruses are often considered the oldest viruses to be studied in the history of science [8]. While HSV-1 is estimated to infect up to one-third of the world population, HSV-2 infects nearly 500 million people around the globe with more than 20 million new cases occurring every year [4]. Importantly, HSV-1 is the foremost important cause of infectious blindness in developed countries and has gained importance in primary genital infection, surpassing in many cases HSV-2 [9–22]. Nevertheless, because HSV-2 recurs significantly more often than HSV-1 in the genitalia, HSV-2 remains overall the most frequent cause of genital ulcers worldwide [23–26]. It is important to bear in mind that HSV-1 and HSV-2 also produce several other pathological conditions, such as encephalitis, conjunctivitis, zosteriform skin lesions, pneumonia, and systemic infections that compromise vital organs [1]. An important concern regarding genital infection with HSV is its association with increased HIV infection. Indeed, genital infection with HSV has been suggested to increase up to 3-4 times the susceptibility of acquiring HIV [27–29], which has been proposed to be mediated, at least in part by soluble mediators at the infection site [30, 31]. Furthermore, individuals coinfected with HSV and HIV shed significantly more these viruses than individuals with single viral infections [32–34].

Important efforts have been invested in the past 20 years on the development of a vaccine against HSVs. However, potential vaccine formulations that have reached the clinic have proven ineffective at preventing infection or reducing virus shedding [35, 36]. Discouraging results derived from the latest HSV-2 vaccine clinical trial, which used a viral subunit formulation, have led to new debates in the field and rethinking on the role of neutralizing antibodies in protecting against HSV-2, as well as the need for correlates of protection [37–39]. Indeed, somewhat unexpected results were obtained with a subunit vaccine consisting of HSV-2 glycoprotein D (gD) plus an adjuvant, which was found to be more efficacious against HSV-1 than against HSV-2-induced genital disease [40, 41]. Again, these data are leading to new paradigm shifts in the field that hopefully will translate into novel vaccine approaches that could eventually reach the clinic. The lack of an effective vaccine against HSVs has flourished onto the development of novel microbicides against these viruses [1].

HSV establishes a lifelong infection in the host by infecting neurons and persisting latently inside these cells [42, 43]. Because sensorial nervous termini innervate the skin and mucosae, infections at these sites with HSVs can lead to neuron infection with a significantly high frequency [44, 45]. Indeed, HSVs can readily gain access to neurons somewhat early after infecting epithelial cells, because these cells interact closely. Nevertheless, to restrict virus access to neurons and other tissues, the host has evolved an arsenal of antimicrobial determinants that aim at blocking infection, progression of infection, and microbe replication. However, as masters of immune evasion, HSVs encode molecular determinants that promote their stealth and overcome host defenses by overriding several of the antiviral elements of the host.

## 2. HSV Infectious Cycle 

Herpes simplex viruses are enveloped viruses with numerous proteins and glycoproteins embedded on their exterior; whether 11 of the viral glycoproteins encoded by the viral genome are present on the virion surface remains to be thoroughly defined [46, 47]. Nevertheless, at least five viral glycoproteins have been implicated in viral entry: glycoprotein B (gB), gC, gD, gH, and gL [48, 49]. gB acts both as a viral attachment protein and fusion protein by binding to heparan sulfates (HS) on the surface of susceptible host cells [50] and also is known to bind to paired immunoglobulin-like type 2 receptor (PILR) alpha [51, 52]. A similar function has been described for gC in virus attachment, although only for HSV-1 [53]. After gB-mediated attachment, gD binds to either of its receptors: nectin-1 (*PVRL1*;* poliovirus receptor-related 1*) expressed on the surface of most host cells or alternatively HVEM (*Herpesvirus Entry Mediator*,* TNFRSF14*), mainly expressed on immune cells [54, 55]. Furthermore, 3-*O*-sulfate HS has also been suggested as a potential receptor for gD, although its physiological relevance requires additional research [56]. Binding of gD to its receptors is thought to induce conformational changes leading to the functional activation of a complex formed by gH/gL [57]. Activated gH/gL complex would in turn then promote changes in gB that activate the fusogenic properties of this protein and mediate the fusion of viral and host cell membranes [58, 59]. As an alternative pathway, HSVs can enter cells through endocytic vesicles [60, 61]. In both cases, fusion of membranes promotes the entry of the capsid and accompanying viral proteins (tegument) into the cytoplasm [62]. The tegument is a complex mesh of >20 proteins beneath the envelope that wraps the viral capsid and contains molecular determinants that mediate, among others, the inhibition of cellular translation and apoptosis [62, 63]. Once released into the cytoplasm, the capsid associates with microtubules through two tegument proteins VP1-2 (encoded by* UL36*) and UL37 and then travels to the outer nuclear membrane to bind to the host nuclear pore complex (NPC) to release the viral DNA into the nucleus [62, 63]. Nucleoporin Nup358 has been associated with this process by docking VP1-2 onto the nuclear pore complex and facilitating the release of viral DNA into the nucleus through this macromolecular complex [64]. Once released into the nucleus, the viral DNA is transcribed by means of the host RNA-polymerase II activity [65, 66]. However, not all HSV genes are expressed synchronously but instead in four consecutive rounds of transcription. First, immediate early genes (alpha) are transcribed, many of which encode for proteins contributing to immune evasion and work as factors controlling cell translation [67]. Then, follows the transcription of early genes (beta) that are required for DNA replication [68]. Finally, early late and late genes (gamma-1 and gamma-2) are transcribed, which mainly encode for structural components of virions, such as capsid, tegument, and surface proteins [69, 70]. These proteins can work as well as important immune evasion determinants (see below). To generate new virions, capsid proteins migrate from the cytoplasm into the nucleus to assemble with viral DNA and acquire at this location a layer of tegument proteins. Unlike other viruses, HSVs do not alter nuclear pores on exit but rather undergo envelopment in the inner nuclear membrane to form an enveloped capsid [71]. The capsid then travels through the perinuclear space and immediately fuses with the outer nuclear membrane thanks to glycoproteins gB and gH, exposing a tegument-recovered capsid into the cytoplasm [72]. Once in the cytoplasm, the capsid is further coated with additional tegument proteins and is once again enveloped in the* trans*-Golgi network [73]. From here, virions are exported in vesicles to the cell surface and secreted. Although host tetherin (Bst-2 or CD317) has been shown to block the release of certain enveloped viruses from the cell surface, the HSV protein vhs (virion host shutoff protein, UL41) can counteract the antiviral function of this protein by depleting it [74]. Noteworthy, HSVs can also propagate directly onto adjacent cells through cell-cell interactions. In these circumstances, virus components are directed to the interface of cell-cell regions. This type of infection is used by HSVs to infect T cells, which has been shown to occur through infected fibroblasts* in vitro* [75, 76]. This type of infection is mediated through a process called virological synapse and provides the virus a safe haven from neutralization by antibodies or complement (see below) [77, 78].

## 3. Evasion of HSV Sensing by Host Receptors

Immune and nonimmune host cells express an array of surface and intracellular receptors intended to sense microbial elements and initiating local and systemic antimicrobial responses. Such receptors, termed pathogen recognition receptors (PRRs), recognize Pathogen Associated Molecular Patterns (PAMPs), which consist among others of microbe-derived molecules, such as lipids, proteins and nucleic acids [79]. An important family of PRRs is Toll-like receptors (TLRs), which upon binding with microbe elements lead to intracellular signaling cascades that promote early antiviral cellular responses and the secretion of soluble mediators that activate infected and noninfected neighboring cells, as well as the immune system [80–84].

**Figure 1:**
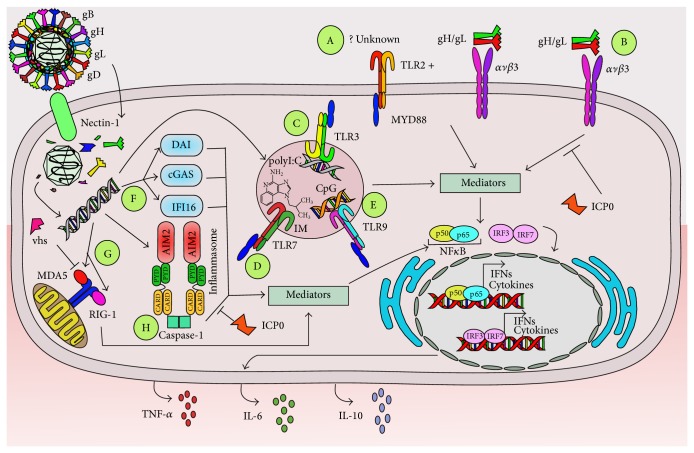
HSVs interfere with host detection of viral determinants. Immune and nonimmune cells express an array of pathogen recognition receptors intended to detect microbes, which ultimately lead to NF-*κ*B and IRF translocation into the nucleus and secretion of antiviral molecules, such as interferons and cytokines. A: Host cells can sense HSV determinants through TLR2, although the specific viral elements detected by this receptor are currently unknown. Intracellular signaling through TLR2 can occur with the help of integrin *ανβ*3-binding after this receptor binds with the HSV complex gH/gL. B: Alternatively, integrin *ανβ*3 can signal intracellularly on its own after gH/gL binding. This process can be interfered by ICP0 C: TLR3, D: TLR7, and E: TLR9 engagement by activating ligands, such as polyI:C, imiquimod (IM), and CpG-ODN, respectively, have been shown to play favorable roles against HSV infection by inducing activating pathways within cells that lead to the secretion of antiviral molecules. F: Nucleic acids generated during HSV infection can also be detected by host intracellular sensors, such as DAI, cGAS, and IFI16. G: The RIG-1/MDA5 complex can also detect virus-derived nucleic acids; however its function is blocked by viral vhs. H: Finally, the inflammasome is activated by HSV determinants, although HSV ICP0 can counteract its activity and negatively modulate its function.

It has been shown that HSVs induce the activation of TLR2 in primary vaginal epithelial cells and also immune cells, such as dendritic cells (DCs). Interestingly, it has been suggested that in keratinocytes, neural cells, and epithelial cells TLR2-mediated effects after virus infection require the cooperation of *ανβ*3-integrin, likely due to the binding of the HSV gH/gL complex to this integrin, leading to NF-*κ*B activation, interferon production, and IL-10 secretion (Figure 1) [85, 86]. In DCs, the activation of TLR2 induces a cell response that leads to the downstream activation of NF-*κ*B and the transcription of immunomodulatory cytokines, such as IL-6, IL-8, IL-10, IL-12, and TNF-*α* [87]. Another study with DCs also showed that HSV recognition by TLR2 promoted IL-6 and IL-12 secretion and proposed that this cytokine outcome was mediated, at least in part, by TLR9 modulation, suggesting a previously uncharacterized mechanism for sequential recognition of viruses via TLR2 through TLR9 [88]. Consistent with this notion, TLR2^−/−^ knockout mice secrete low levels of MCP-1, a chemokine generally induced after TLR9 engagement [89]. Importantly, TLR2^−/−^ knockout mice display prolonged survival after HSV infection, as compared to wild-type and TLR4^−/−^ knockout mice. Despite reduced mortality, viral loads remained similar in TLR2-knockout and wild-type animals [90]. On the other hand, microglia from TLR2^−/−^ mice display delayed and attenuated production of reactive oxygen species (ROS) following viral infection and suffer lesser neuronal oxidative damage in mixed neural cell cultures, as compared to HSV-infected cells from wild-type animals [91].

TLR3 has also been described to play a role in HSV infection, especially for neurons and during viral brain infection. For instance, it has been shown that TLR3 deficiencies (TLR3^−/−^) render astrocytes permissive to HSV infection, facilitating the establishment of CNS infection in animals. Consistently, it was shown that TLR3 expressed in astrocytes provided early control of HSV infection after viral entry into the central nervous system and induced type-I IFN responses in these cells. Remarkably, this TLR3 deficiency did not seem to affect innate immune responses [92]. Other studies have shown that astrocyte infection with HSV leads to TLR3 engagement and NF-*κ*B activation, upregulating the expression of TNF-*α* and IL-6 with antiviral functions attributed to these two molecules (Figure 1) [93]. On the other hand, studies performed in humans carrying mutations that negatively modulate TLR3-mediated immunity have shown that these individuals are more prone to HSV encephalitis [94–96]. Consistent with these findings, a study with* ex vivo* differentiated neurons, astrocytes and oligodendrocytes derived from pluripotent stem cells from individuals with TLR3 deficiencies were shown to be more susceptible to HSV infection* in vitro* than control cells and displayed compromised interferon secretion. Interestingly, these effects depended significantly on the cell types analyzed [97]. On the other hand, mice pretreated either intravaginally or intraperitoneally with agonists for TLR3, such as polyI:C, suffer significantly less virus burden upon intravaginal viral challenge than nontreated animals, suggesting that activating this pathway would play favorable roles against HSV infection (Figure 1) [98].

As with TLR3, pretreating animals with TLR7 agonists, such as imiquimod, has also been shown to significantly reduce HSV burden in the genital tract after viral infection (Figure 1) [99]. Because of these results, imiquimod has been tested in humans, particularly against HSV strains that are resistant to acyclovir in immunocompromised patients, with favorable results [100–102]. However, it is important to note that another study found that imiquimod produced IFN-independent anti-HSV effects in nonimmune cells, which was independent of TLR signaling and IFN production, suggesting that TLR7 is likely not the only activation pathway involved in the favorable results observed against HSV in other studies [101].

TLR9 has also been shown to play roles in HSV infection, although similar to TLR3 and TLR7, because TLR9 agonists can positively influence the antiviral response against this pathogen. Indeed, mice pretreated intranasally with TLR9 agonists, such as CpG-oligodeoxynucleotides (CpG-ODNs), show reduced secretion of inflammatory cytokines, such as CCL2, IL-6, and CCL5, and reduced viral loads in the brain, resulting in mild encephalitis and increased survival rates, as compared to nontreated mice (Figure 1) [103]. Additionally, treatment with CpG-ODN containing unmethylated CpG provides protection against lethal vaginal challenge with HSV that was probably mediated by an intricate crosstalk between plasmacytoid DCs (pDCs) and vaginal stromal cells, as well as type-I IFNs [104–106]. Similar to the findings described with TLR3 and TLR7, these results suggest that modulating TLR9 signaling could be a promising strategy for limiting HSV infection in the host, although results from another group suggest that the antiviral effects mediated by TLR9 antagonists might not necessarily be mediated uniquely by intracellular events linked to TLR9 signaling [107]. Nevertheless, the results obtained with TLR9 agonists suggest that activating this TLR might be a useful strategy for controlling pathological responses induced by HSVs [108]. When combined with antivirals, such as acyclovir or anti-inflammatory molecules, this strategy could improve current therapies against these viruses [103, 109].

Importantly, HSVs also induce the activation of non-TLR sensors in target cells. Namely, primary vaginal epithelial cells display increased activation of DNA sensors, such as DAI (DNA-dependent activator of interferon) and IFI16 (interferon-inducible 16), which trigger the secretion of IL-6 (Figure 1) [110]. Another non-TLR host sensor includes *αvβ*3-integrin, mentioned above with TLR2, which was also recently described to function as a major sensor of HSVs* per se* and activator of innate immunity by relocating the viruses' nectin-1 receptor to cholesterol-rich microdomains, thus, enabling virus uptake into dynamin 2-dependent acidic endosomes [111]. *αvβ*3-integrin interacts with HSVs gH/gL complexes and is thought to signal at least through two pathways, one mediated by TLR2 with the activation of NF-*κ*B and consequently induction of type-I interferons and another involving sarcoma- (SRC-) spleen tyrosine kinase- (SYK-) caspase recruitment domain-containing protein 9- (CARD9-) TRIF (TIR-domain-containing adapter-inducing interferon-*β*), which affects interferon regulatory factor 3 (IRF3) and IRF7 (Figure 1) [85, 86]. Importantly, the HSV viral protein ICP0 can counteract these *αvβ*3-integrin signaling pathways to impair sensing of HSVs by infected cells [86]. A recent study suggests that TLR signaling via MyD88 and TRIF is expendable for controlling HSV infection and spread. Indeed, MyD88^−/−^, TRIF^−/−^, and MyD88^−/−^-TRIF^−/−^ double knockout mice displayed similar levels of HSV replication, when compared to wild-type mice, although this particular study was focused on HSV corneal infection. Importantly, the DNA sensor IFI-16/p204 was identified here to be key for the activation of IRF3 and IFN-*α* production for viral containment [112]. Consistently, silencing the genes that encode for IFI16/p204 inhibits the activation of IRF3 and NF-*κ*B in response to HSV DNA [113]. Furthermore, a recent study showed that IFI16 depletion was associated with increased HSV yield, while its overexpression reduced the amount of virus obtained in cell cultures. ChIP assays found that IFI16 binds to HSV promoters and that cells devoid of this protein display increased amounts of host proteins that promote viral gene transcription at these locations. These findings suggest that IFI16 possesses antiviral functions and negatively modulates HSV transcription after binding to viral DNA [114]. Another intracellular, non-TLR receptor involved in the detection of HSV determinants is cyclic guanosine monophosphate-adenosine monophosphate (cGAMP) synthase (cGAS), a novel cytosolic DNA sensor (Figure 1). This protein has been shown to detect HSV DNA leading to type-I IFN (IFN-I) production in fibroblasts, macrophages, and dendritic cells and mice deficient for cGas succumb to death after infection with this virus [115]. Intracellular nucleic acid sensors RIG-I and MDA5 (retinoic acid-inducible gene 1 and melanoma differentiation-associated protein 5) also play antiviral roles in infected cells, yet vhs can selectively inhibit the expression of these molecules and prevent downstream IRF3 dimerization, as well as the translocation of this complex into the nucleus (Figure 1). By doing so, vhs can block signaling mediated through non-TLR pathways [116].

Another mechanism by which host cells can sense and initiate antiviral responses is through the activation of the inflammasome. The inflammasome is a multiprotein complex involved in translating pathogen recognition events into the secretion of inflammatory molecules, such as IL-1*β*. Relevant inflammasome sensors include NLRP3 and AIM2 in the cytoplasm of cells and the nuclear sensor IFI16, discussed above. Recent studies have shown that HSV can induce early activation of the inflammasome and then, later on, inhibit its function during active infection [117]. Indeed, fibroblasts infected with HSV display activated IFI16 and NLRP3 and secrete IL-1*β* early after infection, although later on IFI16 is targeted to the proteasome by ICP0, likely releasing the break that IFI16 imposes on the transcription of HSV genes [114, 117]. Subsequently, NLRP3 and AIM2 remain unaltered in cells with an inhibited inflammasome and little secretion of mature IL-1*β* [117].

## 4. Modulation of Cell Viability and Early Antiviral Response

**Figure 2:**
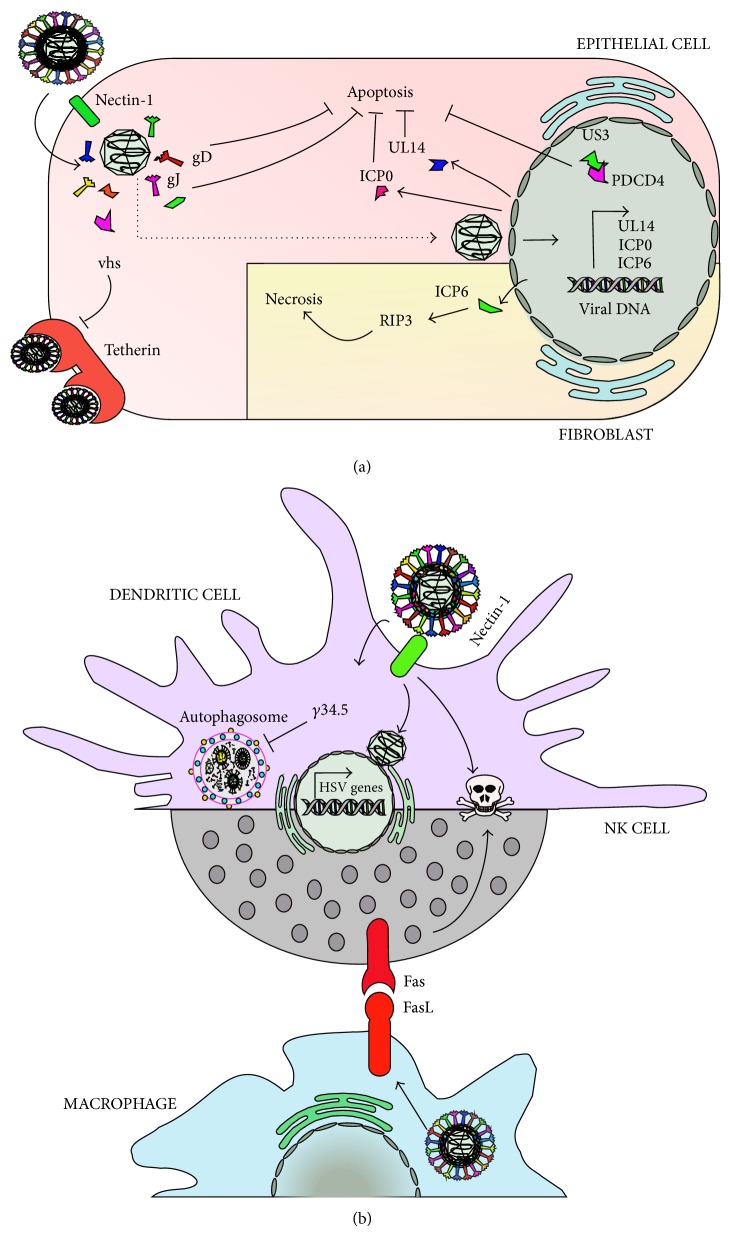
HSVs interfere with cell viability. HSVs encode determinants that modulate cell viability. (a) Within epithelial cells, HSV can extend the survival of cells by blocking apoptosis thanks to viral proteins, such as gD, gJ, UL14, ICP0, and US3. Tetherin, a host antiviral factor involved in blocking virus release from the surface of infected cells, is blocked by HSV vhs. Contrarily, fibroblasts display necrosis upon infection with HSV, which would be mediated by ICP6. (b) Dendritic cells display apoptosis early after infection with HSVs by unknown viral determinants. The autophagosome, which mediates virus control in these and other cells, such as neurons, is inhibited by the viral protein *γ*34.5. HSV also induces apoptosis of NK cells, albeit through Fas/FasL interactions between these cells and HSV-infected macrophages.

Sensing of microbial components by immune and nonimmune cells can lead to cell apoptosis, as a host strategy to block virus replication and spread within cells. Importantly, HSVs encode viral determinants that block or delay the onset of apoptosis in infected cells, likely as a mechanism to extend the viability of its substrate for replication. This process has been proposed to be mediated by viral glycoproteins such as gJ and gD, as virus mutants lacking each one of these proteins initiated apoptotic cascades in epithelial cells (Figure 2(a)) [118]. Furthermore, the viral proteins ICP10PK and UL14 have also been shown to prevent apoptotic processes triggered in neurons and epithelial cells after viral infection (Figure 2(a)) [119–121]. Finally, US3 an HSV protein kinase conserved throughout alphaherpesviruses has also been shown to play a key role in blocking apoptosis induced by viral gene products and exogenous agents in epithelial cells. The antiapoptotic effects of US3 would be mediated through its interaction with programmed cell death protein 4 (PDCD4), which is retained in the nucleus of infected cells (Figure 2(a)) [122].

Nevertheless, other HSV proteins have been proposed to induce apoptosis and necrosis in host cells. For instance, HSV has been shown to induce necrosis in mouse fibroblast cells (L929 cells) mediated by the interaction between the viral ribonucleotide reductase large subunit ICP6 and RIP3 (receptor-interacting kinase 3) through RHIM domains, which activate MLKL (mixed lineage kinase domain-like protein). Consistently, an HSV ICP6 deletion mutant failed to cause effective necrosis of HSV-infected cells and mice lacking RIP3 exhibited severely impaired control of HSV replication and pathogenesis, highlighting the importance of the latter in limiting virus pathology [123]. Noteworthily, another study showed that early after HSV infection, natural killer cells (NK cells) suffer apoptosis through Fas/FasL when these cells interact with HSV-infected macrophages (Figure 2(b)) [124].

Similarly, HSV infection of dendritic cells induces apoptosis early after virus entry, particularly after the release of immunomodulatory cytokines [125, 126], and the viral protein *γ*34.5 can interfere with DC autophagosome maturation, which is thought to play antiviral functions in these cells by degrading virus determinants (Figure 2(b)) [127, 128]. A similar role for autophagy has been proposed in the context of HSV infection in neurons as an alternative to IFN responses, which would likely result in the death of these cells or detrimental outcomes for the host. Indeed, neurons from dorsal root ganglia require autophagy to limit HSV replication* in vivo* and* in vitro* [128].

**Figure 3:**
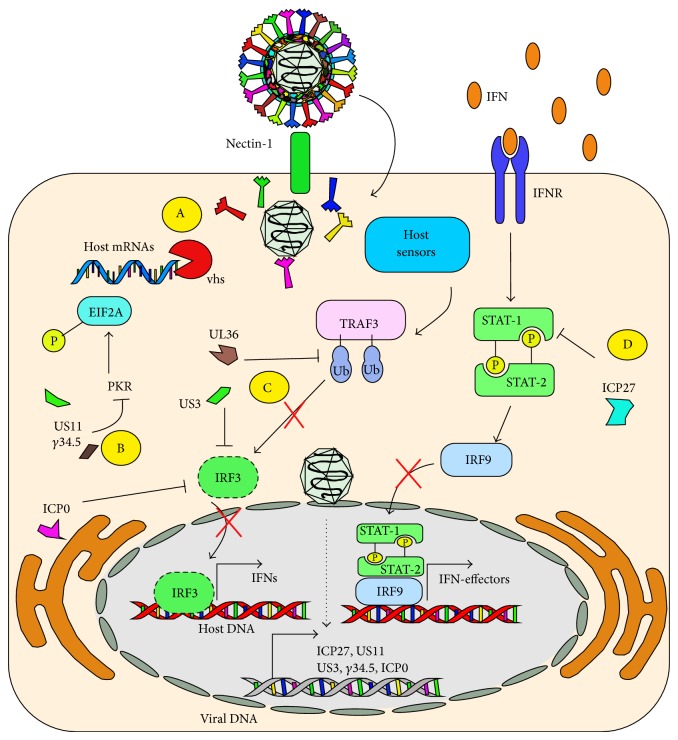
HSVs interfere with the induction of type-I interferons and interferon signaling. A: HSV vhs proteins interfere with cell protein translation by specifically degrading host mRNAs. B: Viral US11 and *γ*34.5 interfere with host PKR function, by impairing its capacity to phosphorylate EIF2A, which blocks translation within infected cells. C: Host sensors activate TRAF3 after detecting HSV determinants, which normally leads to IRF3 activation. However, UL36 interferes with TRAF3 ubiquitination blocking its IRF3-activating capacity. ICP0 and US3 also interfere with IRF3 activation. Impaired IRF3 function will result in poor secretion of interferons by infected cells. D: Viral ICP27 interferes with STAT-1 signaling mediated by IFNR, which would otherwise lead to the secretion of antiviral effectors.

Antiviral functions in host cells are also mediated by Protein Kinase R (PKR), which phosphorylates the translation initiation factor EIF2A as a mechanism to inhibit the translation of RNA messengers upon viral infections. Importantly, this host protein has been shown to play a key role in controlling HSV replication* in vitro* and* in vivo* [129, 130]. However, HSVs have evolved molecular determinants that negatively modulate PKR function. For instance, viral *γ*34.5 and US11 have been proposed to inhibit the activity of PKR to promote the translation of viral proteins (Figure 3) [131, 132]. Furthermore, HSVs have evolved determinants that preferentially block the translation of host molecules over viral genes, in such a way to impair their antiviral activity. Indeed, HSVs vhs protein can mediate the degradation of host messenger RNA through their ribonuclease activity (Figure 3) [133]. The spatial-temporal delivery of vhs has evolved in such a way to display optimal activity early after infection for hampering host mRNA transcription and not to alter viral mRNAs transcribed later on [134].

Early sensing of viral determinants by host PRRs will generally lead to the activation of interferon pathways that aim at impairing virus replication and its shedding within the host [135]. Although cells in the genital tract infected with HSV-2 produce interferons in response to this virus, the magnitude of this response is generally hampered in the infected tissue, suggesting that these molecules likely play favorable antiviral roles [136]. Indeed, biopsies obtained from individuals infected with HSV show extremely low levels of type-I IFNs (IFN-*α* and IFN-*β*), despite the presence of a large number of cells capable of synthesizing these mediators, which suggests alterations in the host interferon response during HSV infection [137]. Consistent with this notion, type-I IFN receptor (IFNAR) knockout mice inoculated in the footpads with HSV manifest systemic viral infections that affect the lungs, liver, and spleens, although disease is nonlethal [138]. Interference with host interferon pathways would be mediated, at least in part by the early viral protein ICP0, which can impair IRF3 function and block the transcription of genes regulated by this transcription factor [137]. Additionally, HSV ICP27 also inhibits type-I IFN signaling and interferes with nuclear accumulation of STAT-1 (Figure 3) [139]. Furthermore, the HSV Ser/Thr kinase US3 can hamper IFN-*β* production by hyperphosphorylating IRF3 and by blocking the dimerization and nuclear translocation of this factor (Figure 3) [140]. Similarly, the tegument protein VP16 can also abrogate IFN-*β* expression by inhibiting NF-*κ*B and IRF3 activation by impairing the recruitment of the coactivator CBP, without interfering with IRF3 dimerization, nuclear translocation, or its DNA binding activity (Figure 3) [141]. Yet, another mechanism by which HSV inhibits IFN-*β* expression is through the deubiquitination of TRAF3 by the viral ubiquitin-specific protease UL36, which inhibits stimuli-induced IRF3 dimerization, promoter activation, and the transcription of IFN-*β* (Figure 3) [142]. As noted, HSVs have evolved redundant and nonredundant mechanisms to specifically impair the function of host molecules that are key for the expression of antiviral molecules, namely, interferons.

The importance of IFNs in controlling infection by HSVs is highlighted by the fact that the frequency of genital herpetic recurrences can be reduced in patients by applying topical IFN-*α*, which also reduces viral dissemination [143]. Moreover, the recently described interferon IFN-*ε*, characterized as a type-I IFN constitutively expressed by epithelial cells in the female and male reproductive tract, is proposed to be a potent antiviral host mediator that likely contributes to control of HSV infection [144, 145]. However, the exact mechanism by which IFN-*ε* exerts its anti-HSV effects remains to be determined. Importantly, expression of IFN-*ε* varies with the female hormonal cycle and seems to be limited to cells belonging to reproductive organs [145, 146].

## 5. Secretion of Immunomodulatory Mediators Early after HSV Infection

After HSV has blocked immediate host antiviral responses, which rely on the sensing of microbe elements and early interferon responses, cell damage resulting from virus replication likely spreads virus-elicited danger signals and damage-associated molecular patterns (DAMPs) onto other noninfected cells [117]. These neighboring cells, as well as patrolling immune cells, may sense these danger elements and initiate cytokine and chemokine responses that will modulate the* milieu* and other immune components [126, 147, 148]. Whether the soluble mediators produced in response to these danger signals or HSV itself promote the clearance of the virus or favor its persistence and spread in the host is largely unclear.

Because cytokine secretion is generally dependent on the canonical activation of NF-*κ*B, HSVs have evolved several molecular mechanisms to modulate the activity of this transcription factor. A recent report showed that the viral DNA polymerase processivity factor UL42 interacts with p65/RelA and p50/NF-*κ*B1 to block the translocation of NF-*κ*B to the nucleus in response to stimuli, such as TNF-*α* [149]. Consistently, another study found that HSV ICP0 inhibits TNF-*α*-induced NF-*κ*B activation, interacting similarly with p65/RelA and p50/NF-*κ*B1 [150]. US3 has also been shown to significantly inhibit NF-*κ*B activation and decrease the expression of inflammatory chemokines, such as IL-8 [151]. Furthermore, HSV VP16 also inhibits NF-*κ*B activation and blocks IFN-*β* production [141]. However, other HSV proteins, such as tegument protein UL37, have been shown to promote NF-*κ*B activation and IL-8 secretion in keratinocytes [152]. Activation of NF-*κ*B after cell infection has also been reported to facilitate viral replication [153, 154]. Taken together, HSVs have evolved strategies to both block and promote the activation of NF-*κ*B within infected cells. Whether these opposing effects depend on the cell types targeted by these viruses or different stages of the infectious cycle requires further study. Nevertheless, these findings highlight the importance of NF-*κ*B modulation by HSVs after infection.

Despite interference with NF-*κ*B activity, cells infected with HSV nonetheless secrete numerous modulatory cytokines and chemokines after infection or at the site of inflammation. For instance, HSV has been shown to induce the secretion of CCL2, IL-8, IL-6, and TNF-*α* in primary endometrial genital epithelial cells [155].* In vivo*, HSV promotes CXCL9 expression in the cervical mucus of HSV-positive women [156]. Importantly, this chemokine and CXCL10 have been shown to play important roles against HSV in CNS infection in the mouse model, likely by recruiting NK and cytotoxic T cells to the infected tissue [157]. Similarly, a recent study proposed that CXCL10 is needed for establishing protective immunity against HSV-2 genital infection after vaccination with an attenuated HSV strain [158]. CCL2 induced upon HSV infection has been attributed a favorable role in corneal infection in the mouse model. Indeed, CCL2^−/−^ mice were unable to contain the virus and failed to recruit inflammatory monocytes to the infection site [159]. Furthermore, CCL2 expression driven by IFI16 recognition of HSV has been described to facilitate the recruitment of inflammatory monocytes to the infection site, as silencing of p204/IFI-16 resulted in the loss of CCL2 production and significantly more HSV shedding [159]. As indicated above, another cytokine induced after HSV infection is IL-6. Noteworthily, a protective role has been attributed to this cytokine in microglia, likely through downstream signaling of Signal Transducer and Activator of Transcription 3 (STAT3), although the precise mechanism leading to its protective role is unclear [160, 161]. Mast cells have also been shown to secrete IL-6 early after HSV infection, as well as TNF-*α*, yet these cytokines were not induced directly by HSV in this study but depended on supernatants from HSV-infected keratinocytes and the IL-33 receptor on the former cells. Importantly, mice lacking TNF-*α* or IL-6 succumbed to death, consistent with a protective role for these cytokines [162]. Contrarily, a recent study suggested that treating mice with anti-TNF-*α* in combination with the antiviral valacyclovir could significantly improve the prognosis of encephalitis caused by HSV [109].

One aspect that has brought important attention onto cytokines and chemokines produced after HSV infection is that some of the molecules secreted in the genital tract can favor host infection by other sexually transmitted pathogens, such as the human immunodeficiency virus (HIV) [30]. Indeed, infection with HSV-2 increases 3-4 times host susceptibility of acquiring HIV and, furthermore, coinfection increases the shedding of both viruses [27–29, 163]. These findings have been corroborated in a murine infection model [31]. The increased susceptibility to acquire HIV after HSV-2 infection has been suggested to result, within others, by an increased recruitment of target cells for HIV, such as dendritic cells and T cells to the site of infection [164, 165], increased expression of HIV-receptor molecules at the surface or specific cell populations [166, 167], and reduced expression of cell surface molecules that actually promote the capture and degradation of HIV [168]. Furthermore, immune cells such as dendritic cells infected with HSV have been shown to produce soluble mediators that promote the reactivation of HIV from cells latently infected with the latter virus [126]. Regretfully, the identities of the soluble molecules that account for the observed effect have not been identified so far.

Besides upregulating the expression of certain cytokines and chemokines, HSVs can also reduce the expression of certain antiviral molecules, such as the secreted leucocyte protease inhibitor (SLPI). Indeed, HSV-infected cells secrete less SLPI, which reduces the infectivity of HSV* in vitro* [169]. Furthermore, a decrease in the expression of SLPI would likely promote an increase in the secretion of proinflammatory cytokines, which are associated with exacerbated damage to the infected tissues [169]. Nevertheless, virus-mediated inhibition of other chemokines could favor the host, such as CXCL2 which is secreted by monocytes in response to HSV which is known to recruit neutrophils that elicit damaging inflammatory immune responses to host cells and tissues, namely, neurons [170].

Despite the fact that HSVs have been extensively studied, it is surprising to note how little we know about the contribution of cytokines and chemokines induced upon infection by this virus to infection and pathology. Cytokines and chemokines induced by HSV infection will likely play different roles for the host, either favorable or antagonizing, depending on the tissue infected, whether it is skin, genitalia, eyes, or the central nervous system. Furthermore, differences in the nature and amounts of the cytokines and chemokines secreted upon viral infection, as well as the roles of these molecules on virus clearance and disease, will likely depend on whether infection is mediated by HSV-1 or HSV-2. High throughput techniques such as multiplex cytokine arrays and the availability of numerous knockout mice for these molecules should provide new insights and valuable information on the role of these soluble mediators in HSV infection in the near future.

## 6. HSV Interferes with Innate Immune Functions 

Innate immunity has evolved soluble components and specialized cells to block microbe infection, replication, and shedding. One such element is the complement. The complement is composed of serum proteins that, once triggered by microbial determinants or antibody bound to ligands, interact with each other in a cascade of events that lead to the damaging of the surfaces of the pathogen or cells infected with the microbe. However, HSVs have evolved molecular determinants to interfere with the function of complement protein C5 and block its downstream activating properties; this process is mediated by glycoprotein C [78, 171]. By doing so, the virus likely extends its lifespan in the serum and that of the cells it infects.

NK cells play important roles against several viral pathogens; however their role in HSV infection is frequently debated. Although some studies propose key roles for these cells, others have underestimated their importance at controlling HSV infection [172, 173]. HSV can directly activate NK cells through TLR2; whether this interaction promotes viral clearance or not* in vivo* is still unclear [174]. HSV can also decrease the expression of NK-activating ligands such as MICA (MHC class I polypeptide-related sequence A) on the surface of infected cells, thus interfering with the effector activity of these cells. This process would be mediated by a late HSV gene product, which would mask, internalize, or retain MICA intracellularly [175, 176].

Natural killer T (iNKT) cells are CD1d-restricted T cells that express invariant TCR chains, as well as NK surface markers, and are specialized in recognizing polar lipids presented on the surface of CD1d molecules [177]. Although variations can be observed in the amount of iNKT cells present after HSV infection, changes in the number of cells and expression of markers on their surface are somewhat discrete when compared to patients in the steady state, suggesting potential modulation of these cells by HSVs [178]. Furthermore, infection with HSV has been described to negatively modulate the activity of NKT cells by simply directing CD1d molecules from the cell surface of infected cells into intracellular compartments, thus blocking antigen presentation [179, 180].

## 7. Concluding Remarks

World prevalence for HSVs is a truthful testimony of the success of these viruses in establishing latent infection in the host. Successful infection with HSVs is likely the result of a wide array of viral determinants encoded by these viruses with the capacity to interfere with multiple host factors intended to control early infection by pathogenic microbes. Indeed, HSVs effectively block early cellular antiviral mechanisms by extending the survival of cells that serve as substrates, hence favoring virus production and killing cells that initiate and modulate effective antiviral immune responses, such as DCs. Additionally, these viruses promote their stealth by interfering with their sensing by infected cells and by mounting somewhat modest interferon and cytokine responses that favor their replication and shedding. These phenomena will ultimately allow these viruses to reach cells needed for establishing latency: neurons. Noteworthily, important progress has been made in the last years in identifying early antiviral components elicited and blocked by HSVs. These studies will hopefully lead to the identification and development of drugs that specifically interfere with viral processes. Noteworthily, findings, such as those related to the activation of particular TLRs that favor host responses against these viruses, will undoubtedly contribute to the development of novel antiviral therapies. Indeed, potentiating early antiviral functions in the host before exposure could be as effective as novel anti-HSV microbicides currently under development, while an effective vaccine against these viruses reaches the clinic.
